# Transcription Factor 2I Regulates Neuronal Development via TRPC3 in 7q11.23 Disorder Models

**DOI:** 10.1007/s12035-018-1290-7

**Published:** 2018-08-17

**Authors:** Marielle H. S. Deurloo, Ekaterina Turlova, Wen-Liang Chen, You Wei Lin, Elaine Tam, Nardos G. Tassew, Michael Wu, Ya-Chi Huang, Jacqueline N. Crawley, Philippe P. Monnier, Alexander J. A. Groffen, Hong-Shuo Sun, Lucy R. Osborne, Zhong-Ping Feng

**Affiliations:** 1 Department of Physiology, University of Toronto, 1 King’s College Circle, 3306 Medical Sciences Building, Toronto, ON M5S 1A8 Canada; 2 Department of Functional Genomics, CNCR, Neuroscience Campus Amsterdam, VU University and VU Medical Center, 1081 HV Amsterdam, Netherlands; 3 Department of Surgery, University of Toronto, 1 King’s College Circle, 1184 Medical Sciences Building, Toronto, ON M5S 1A8 Canada; 4 Department of Medicine, University of Toronto, 661 University Avenue, MaRS Centre, 1515 West Tower, Toronto, ON M5G 1M1 Canada; 5 Vision Division, Krembil Research Institute, Krembil Discovery Tower, KDT-8-418, 60 Leonard Street, Toronto, ON M5T 2S8 Canada; 6 MIND Institute, University of California Davis School of Medicine, Sacramento, CA 95817 USA; 7 Department of Molecular Genetics, University of Toronto, 661 University Avenue, MaRS Centre, 1515 West Tower, Toronto, ON M5G 1M1 Canada

**Keywords:** General transcription factor 2i, TRPC3, Cortical neurons, Williams-Beuren syndrome (WBS)

## Abstract

Williams syndrome (WS) and 7q11.23 duplication syndrome (Dup7q11.23) are neurodevelopmental disorders caused by the deletion and duplication, respectively, of ~ 25 protein-coding genes on chromosome 7q11.23. The general transcription factor 2I (*GTF2I,* protein TFII-I) is one of these proteins and has been implicated in the neurodevelopmental phenotypes of WS and Dup7q11.23. Here, we investigated the effect of copy number alterations in *Gtf2i* on neuronal maturation and intracellular calcium entry mechanisms known to be associated with this process. Mice with a single copy of *Gtf2i* (*Gtf2i*^+/Del^) had increased axonal outgrowth and increased TRPC3-mediated calcium entry upon carbachol stimulation. In contrast, mice with 3 copies of *Gtf2i* (*Gtf2i*^+/Dup^) had decreases in axon outgrowth and in TRPC3-mediated calcium entry. The underlying mechanism was that TFII-I did not affect TRPC3 protein expression, while it regulated TRPC3 membrane translocation. Together, our results provide novel functional insight into the cellular mechanisms that underlie neuronal maturation in the context of the 7q11.23 disorders.

**Electronic supplementary material:**

The online version of this article (10.1007/s12035-018-1290-7) contains supplementary material, which is available to authorized users.

## Introduction

The general transcription factor 2I, GTF2I (protein TFII-I), is one of ~ 25 genes encoded within the chromosome 7q11.23 region commonly deleted or duplicated in Williams syndrome (WS) (MIM:194050) and Dup7q11.23 (MIM:609757), respectively. Both syndromes are associated with neurocognitive and behavioral features, with somewhat contrasting phenotypes. WS is characterized by dysmorphic features, elastin arteriopathy, mild to moderate intellectual disability, deficits in visuospatial construction, relative strength in language, and an atypical personality that includes over-friendliness and nonsocial anxiety [1]. In contrast, Dup7q11.23 is associated with speech and language delay, both social and nonsocial anxiety, and autism [2].

Several brain structure abnormalities have been described in individuals with WS (reviewed in [3]), including reduced gray matter volume in the parietal and occipital lobe, altered cell size and density in the primary visual cortex, and a decreased overall curvature of the brain with abnormally increased gyrification. In some individuals with Dup 7q11.23, increased brain volume [4] or cortical dysplasia [5] have been described, and at least half of individuals with Dup7q11.23 have macrocephaly (Morris et al. 2015). These structural abnormalities lead to the question whether neuronal morphology might underlie the observed differences in brain size and cortical structure seen in WS and/or Dup7q11.23.

We previously generated mice with decreased (*Gtf2i*^*+/Del*^) or increased (*Gtf2i*^*+/Dup*^) genomic copy number of *Gtf2i*, a strong candidate for the neurobehavioral features of WS and Dup7q11.23 [6]. Decreased copy number of this gene has been shown to result in increased social interaction and impaired object recognition in mice [7], whereas increased copy number was linked to separation anxiety in both mice and humans [6]. TFII-I is highly expressed in the prenatal and postnatal developing brain [8], regulates gene expression in induced pluripotent stem cells [9] (reviewed in [10]), and modulates the function of serum response factor in neurons [11]. In addition to these nuclear activities, TFII-I also plays a role in the cytoplasm where it acts as a regulator of intracellular calcium levels through interaction with phospholipase C (PLC) γ-isoforms [12]. PLC isoforms γ regulate intracellular calcium levels via intracellular stores, and calcium entry through transient receptor potential (TRP) channel TRPC3. TFII-I competes with TRPC3 for binding to PLC-γ and was suggested to play the role of a negative regulator for TRPC3 membrane targeting, leading to subsequent inhibition of calcium entry [12]. Cytosolic Ca^2+^ is extremely important in axonal and dendritic development (for review see [3]). An optimal level of free [Ca^2+^]_i_ is required for maximal neurite outgrowth and as a result, fluctuation of [Ca^2+^]_i_ levels through specific membrane TRP channels has been linked to changes in neuronal morphology [13, 14] and maturation [15]. The cellular effects of *Gtf2i* deletion and duplication on calcium signaling and neuronal development, however, have not been tested directly.

In this study, we investigated copy number effects of Gtf2i on the maturation of cortical neurons and calcium entry in mutant mice with Gtf2i deletion or duplication. We further studied the differences in TRPC3 channel cellular localization and calcium signaling in the *Gtf2i* mutant mice. We showed that deregulation of TRPC3-mediated calcium levels in the Gtf2i mutant neurons could contribute to their morphological changes. These findings extend the previous behavioral characterizations of mouse models of WS and Dup7q11.23 to level of molecular mechanisms, thus offering valuable clues for future research.

## Materials and Methods

### Animals

All procedures were approved by the University of Toronto Animal Care Committee and performed in accordance with the Canadian Council on Animal Care guidelines. Generation of the *Gtf2i*^*+/Dup*^ and *Gtf2i*^*+/Del*^ mice was described previously [6] and mice were maintained on a CD1 outbred background.

### Primary Dissociated Cultures

Cortical cultures were prepared from embryonic day E17- E18 pups of either sex following the published protocol [16, 17] with modifications. The cortex was dissected from the mouse brain and digested with 0.025% Trypsin/EDTA at 37 °C in HBSS (Hanks balanced salt solution; Sigma, St. Louis, MO, USA) for 15 min and washed with pre-warmed medium to stop digestion. Cells were triturated approximately 10 times with a 1000-μL tip. Neurons were centrifuged at 1000 rpm for 5 min, supernatant was removed, and cells were resuspended in culture media. Cell density was determined using an improved Neubauer hemocytometer and low-density cultures were plated on poly-D-lysine (0.1 mg/ml Sigma) coated glass coverslips (18 mm #1.5, Warner Instruments). Neurons at a density of 100,000 neurons/cm^2^ were maintained in neurobasal medium (Invitrogen, Carlsbad, CA, USA) with 2% B27 supplement, 100 U penicillin, 100 μg streptomycin, and 2 mM GlutaMAX at 37 °C with 5% CO_2_ for 4 to 10 days. Cultured cortical neurons were transfected 4 h after plating with Trpc3-siRNA (20 nM, sc-42667, Santa Cruz) or no siRNA as negative control. In each case, an expression vector encoding eGFP was included to mark cell transfection using Lipofectamine 2000 following the manufacturer’s instruction (Invitrogen #11668019) [17]. All experiments were carried out blind to genotype.

### Immunocytochemistry, Confocal Image Acquisition and Analysis

Mouse cortical neurons were fixed on DIV4, 6, 8, or 10 with 4% paraformaldehyde and permeabilized with 0.1% Triton X-100 in PBS for 20 min at room temperature, and stained and imaged as described previously [15–18]. Day of neuronal culture is counted as DIV0. Primary antibodies: anti-TRPC3 (1:500, Novus Biologicals, #NB110-74935, Littleton, CO, USA); anti-TFII-I (1:50, Santa Cruz, #sc-9943, Santa Cruz, CA, USA); anti-NeuN (1:200, Millipore, #MAB377, Darmstadt, Germany); anti-α-tubulin (1:1000, Sigma, #T-5168); anti-MAP2 (1:500, Milipore, #AB15452); or anti-Tau1 (1:100, Millipore, #MAB3420). Secondary antibodies: Alexa Fluor 488 goat anti-rabbit, Alexa Fluor 568 rabbit anti-goat, Alexa Fluor 405 goat anti-mouse, Alexa Fluor 488 goat anti-chicken (1:500, Molecular Probes, Eugene, OR, USA).

Confocal image z-stacks of fluorescence signals were captured with a Carl Zeiss Confocal Laser Scanning Microscope LSM700 with either × 63 DIC (NA 1.40) or × 40 DIC (NA 1.3) oil immersion lenses. All cells for data collection were imaged at a resolution of 1024 × 1024 pixels using the same magnification and laser settings, as described previously [15, 16, 18]. Fluorescence intensity was measured using ImageJ (NIH, http://rsb.info.nih.gov/ij) and corrected for background measured outside of the cell by subtraction of the average background. The values of total, dendritic, and axonal neurite length were analyzed in Matlab using SynD, a semi-automated image analysis routine [19]. Sholl analysis provides a quantitative measurement counting the number of neurite intersections through concentric circles of gradually increasing radius starting at the cell body. The exact number of branches was counted using the NeuronJ plugin for ImageJ [18, 20]. Experiments were performed blind to genotype and the cortical neurons were imaged and traced independently by two people.

### Subcellular Fractionation Protocol

The cortex from postnatal mice of either sex (P7) was dissected and homogenized in 250 μL subcellular fractionation buffer (250 mM sucrose, 20 mM HEPES (7.4), 10 mM KCL, 1.5 mM MgCl2, 1 mM EDTA, 1 mM EGTA and 1 mM DTT, and 1% protease inhibitor (PI) cocktail). All steps were done at 4 °C. The lysate was passed 10 times through a 25G needle and incubated on ice for 20 min then centrifuged at 800*g* for 10 min after which the supernatant (S1) was removed and placed in a clean tube. The nuclear pellet (P1) was washed by adding 500 μL subcellular fractionation buffer, passed through a 25G needle 10 times, and centrifuged again at 800*g* for 10 min. The supernatant was removed and the nuclear pellet was resuspended in RIPA buffer (plus 1% PI). The supernatant (S1) was centrifuged at 10,000*g* for 20 min and the supernatant (S2) was removed. This is the membrane and cytosolic fraction. To obtain the membrane fraction, S2 was centrifuged at 100,000*g* for 1 h. The supernatant (cytosolic fraction) was removed and placed in a clean tube and the membrane pellet was washed by adding 500 μL of subcellular fractionation buffer and centrifuged again at 100,000*g* for 1 h. After the supernatant was removed, the membrane fraction was resuspended in RIPA buffer (+PI).

### Western Blot

The procedures of Western blot were carried out as previously described [15]. Protein samples (20μg) were separated in an 8% DS-PAGE gel and transferred to a nitrocellulose membrane (#66485; PALL Life Sciences) via semi-dry transfer (350 mA, 90 min). Primary antibodies: Rabbit anti-TRPC3, 1:200, #ab 51560, Abcam; rabbit anti-Pan cadherin, 1:1000, ab6505, #ab16505, Abcam; mouse anti-GAPDH, 1:20,000, #2118S, Cell Signaling Technology. Densitometry was employed to quantify proteins of interest using the gel analyzer function of ImageJ (ver. 1.46a; National Institutes of Health, USA).

### Calcium Imaging

To measure intracellular calcium ([Ca^2+^]_i_) levels, neurons were loaded with 2 μM Fura-2 AM (Molecular Probes, Eugene, OR, USA) in extracellular solution composed of (mM) 140 NaCl, 2 CaCl_2_, 1 MgCl_2_, 10 HEPES, 10 glucose, 4 KCl (pH 7.3–7.4 and 320–330 mOsm) for 30 min at 37 °C, as described previously [13–15, 21]. Ratiometric imaging was performed by alternate excitation at 340 and 380 nm by a Deltaram V single monochromator (PTI) controlled by EasyRatioPro (PTI, Edison, NJ, USA) in a dark environment at room temperature [13–15, 21]. The signals were recorded by an intensified charged-coupled device (ICCD) camera (PTI), and fluorescence intensity (Poenie-Tsien) ratios of images were calculated using EasyRatioPro. Neurons were perfused with extracellular solution containing 100 μM carbachol (CCh, Abcam) in which Ca^2+^concentrations were varied as indicated. Intracellular calcium stores were depleted and blocked by 10 mM caffeine and 1 μM thapsigargin (Tocris).

### Electrophysiological Measurements

Whole-cell patch-clamp recordings (ruptured) were performed on cultured cortical neurons using an Axopatch 200B patch-clamp amplifier and Digidata 1322A (Molecular Devices, Sunnyvale, CA, USA) digitizer, as described previously [13–15, 22]. The external solution contained (mM) 120 NaCl, 5.4 KCl, 1 MgCl_2_, 2 CaCl_2_ 20 HEPES, and 10 glucose (pH 7.4, NaOH)), as well as various blockers including 0.2 μM tetrodotoxin (TTX, Tocris), 200 μM CdCl_2_, 10 μM bicuculline (BCC, Sigma), and 20 μM CNQX (Tocris). The pipette (3–6 MΩ) was filled with the pipette solution containing (mM) 150 CsCl, 2 MgCl_2_, 0.3 Mg-ATP, 0.03 Na_2_-GTP, and 1 EGTA (pH 7.2, CsOH). A voltage ramp (from − 100 to 80 mV, over 300 ms) was applied with a holding potential of − 60 mV. The recordings were performed with or without 100 μM CCh at room temperature (22–25 °C). Clampfit 10.3 (Axon Instrument) and Origin 8.1 (OriginLab) were used for analyses of data.

### Statistical Analysis

All data are presented as the mean ± s.e.m.. Statistical analysis was carried out using Prism5 (GraphPad, La Jolla, CA, USA). Differences between mean values from each experimental group were tested using one-way analysis of variance (ANOVA) followed by Tukey’s multiple comparison test or unpaired *t* test for two groups. Nonparametric Kruskal-Wallis test followed by Dunn’s multiple comparison test was also carried out for nonparametric comparison of the data. Differences were considered significant if *P* < 0.05.

## Results

### *Gtf2i*^*+/Del*^ and *Gtf2i*^*+/Dup*^ Cortical Neurons Have Contrasting Differences in Neurite Length and Axonal Branching

**Fig. 1:**
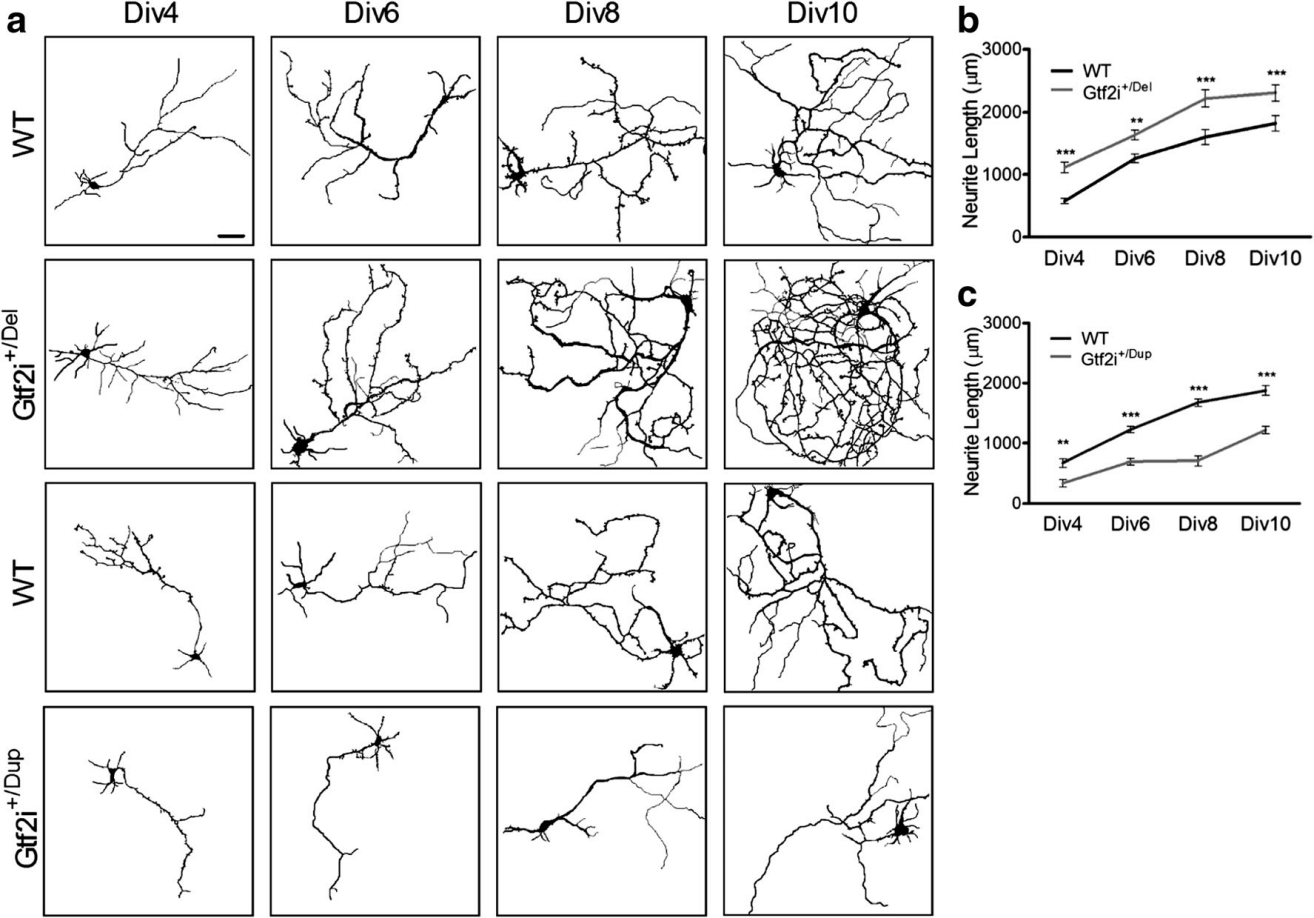
Time course showing total neurite length increase in Gtf2i^+/Del^ and decrease in Gtf2i^+/Dup^ neurons compared to wildtype. **a** Representative images showing neurite length visualized by tracing the neurites at the indicated time-points. **b** Total neurite length per neuron significantly increased in Gtf2i^+/Del^ mice at all time-points (DIV4 *n* = 120; DIV6 *n* = 80; DIV8 *n* = 75; DIV10 *n* = 78) compared to neurons from WT mice (DIV4 *n* = 120; DIV6 *n* = 81; DIV8 *n* = 72; DIV10 *n* = 69). **c** Total neurite length per neuron significantly decreased in Gtf2i^+/Dup^ mice at all time-points (DIV4 *n* = 121; DIV6 *n* = 80; DIV8, *n* = 75; DIV10 *n* = 80) compared to neurons from WT mice (DIV4 *n* = 120; DIV6 *n* = 85; DIV8 *n* = 75; DIV10 *n* = 80). The difference was significant at all time-points and increased from DIV6 onwards. Data are presented as mean ± s.e.m. *P < 0.05, **P < 0.005, ***P < 0.0005. n, number of cells measured in total over 3 separate repeated experiments. One-way ANOVA followed by Tukey’s multiple comparisons test and Kruskal-Wallis followed by Dunn’s multiple comparison test were used for all statistical analysis, except specifically mentioned otherwise. Scale bar 50 μm

The altered brain volume and structure, such as cortical folding abnormalities, reported in both WS and Dup7q11.23 suggest that one or more genes within the critical region affect neuronal development and structure at a cellular level. We studied the correlation between the copy number of Gtf2i and neurite outgrowth patterns in dissociated cortical neurons from E17–E18 Gtf2i^*+/*Del^ and Gtf2i^*+/*Dup^ mice and their wildtype (WT) littermates on DIV 4, 6, 8, and 10 (Fig. 1a). We found that compared to their WT littermates, Gtf2i^*+/*Del^ mice showed a significantly greater total neurite length per neuron at all time-points (*P* < 0.005) (Fig. 1b). In contrast, Gtf2i^*+/*Dup^ mice showed a significantly shorter total neurite length per neuron than their WT littermates (*P* < 0.005) (Fig. 1c).

**Fig. 2:**
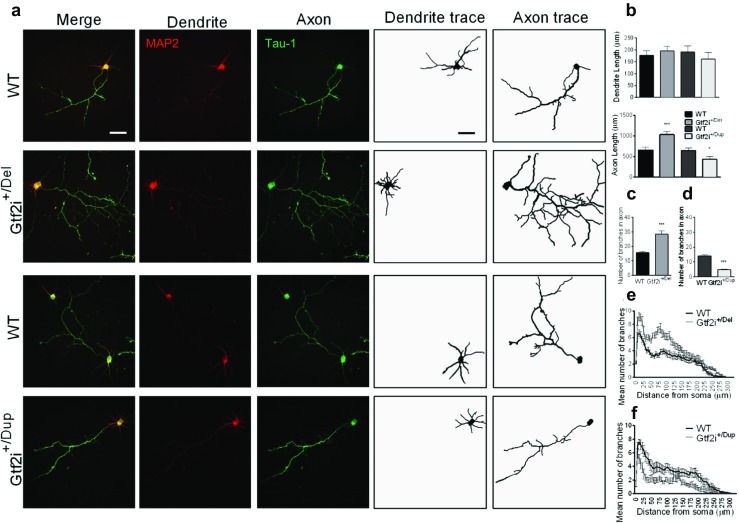
Axonal length and branching increase in Gtf2i^*+/*Del^ and decrease in Gtf2i^*+/*Dup^ neurons. **a** Representative images of neurons immunostained with MAP2 (dendrite marker, red) and Tau-1 (axon marker, green). Neurons were traced separately MAP2 and Tau-1 to measure dendritic and axonal length and branching. **b** Dendritic length of Gtf2i^*+/*Del^ neurons (*n* = 80) and Gtf2i^*+/*Dup^ neurons (*n* = 80) was not significantly different from WT neurons (WT Gtf2i^*+/*Del^ *n* = 80; WT Gtf2i^*+/*Dup^ *n* = 81. *P = 0.72*). **c** Axonal length was significantly increased in Gtf2i^*+/*Del^ neurons (Gtf2i^*+/*Del^ *n* = 81; WT *n* = 80. *P* < 0.0005) and significantly decreased in Gtf2i^*+/*Dup^ neurons (Gtf2i^*+/*Dup^ *n* = 80; WT, *n* = 81, *P* < 0.05). **c**, **e** The total number of axonal branching was significantly increased in Gtf2i^*+/*Del^ neurons (*P* < 0.0005); and **d** reduced in Gtf2i^*+/*Dup^ (*P* < 0.0005) (*n* = 80 cells for each group) as compared to WTs. **e**, **f** Sholl Analysis: the number of axon branches measured at various distance from the soma. **e** Axonal branching was significantly increased in Gtf2i^*+/*Del^ neurons and **f** decreased in Gtf2i^*+/*Dup^ neurons. *P < 0.05, **P < 0.005, ***P < 0.0005

During maturation, one of the key morphological changes of neurons is polarization, as indicated by the emergence of distinct axonal and dendritic processes. In order to examine whether the differences in total neurite length observed in Gtf2i^*+/*Del^ and Gtf2i^*+/*Dup^ cortical neurons originated from the axons or the dendrites, or from both, the length of the axon and dendrite was separately traced at DIV 4 using the markers Tau-1 and MAP2, respectively (Fig. 2a). The dendritic length of Gtf2i^*+/*Del^ and Gtf2i^*+/*Dup^ neurons was similar to that of WT neurons; however, the length of axons was significantly longer in Gtf2i^*+/*Del^ neurons (*P* < 0.0005) and significantly shorter in Gtf2i^*+/*Dup^ neurons (*P* < 0.05) (Fig. 2b). In addition, the numbers of axonal (Tau-1 positive) branches were clearly different among the genotypes. Compared to WT neurons, Gtf2i^*+/*Del^ neurons showed significantly more axonal branching both in the total number of axonal branches (Fig. 2c; *P* < 0.0005) and the number of branches measured at different distances from the soma (Sholl analysis; Fig. 2e). Consistently, Gtf2i^*+/*Dup^ mice showed significantly less branches in both measurements (*P* < 0.0005) (Fig. 2d, f). These findings suggest that *Gtf2i* copy number strongly affects the morphological development of cortical neurons in culture.

### Gtf2i^*+/*Del^ Neurons Have Increased Membrane Expression of TRPC3 Channels, in Contrast to the Widespread Cellular Distribution of the Channels Seen in *Gtf2i*^*+/Dup*^ Neurons

**Fig. 3:**
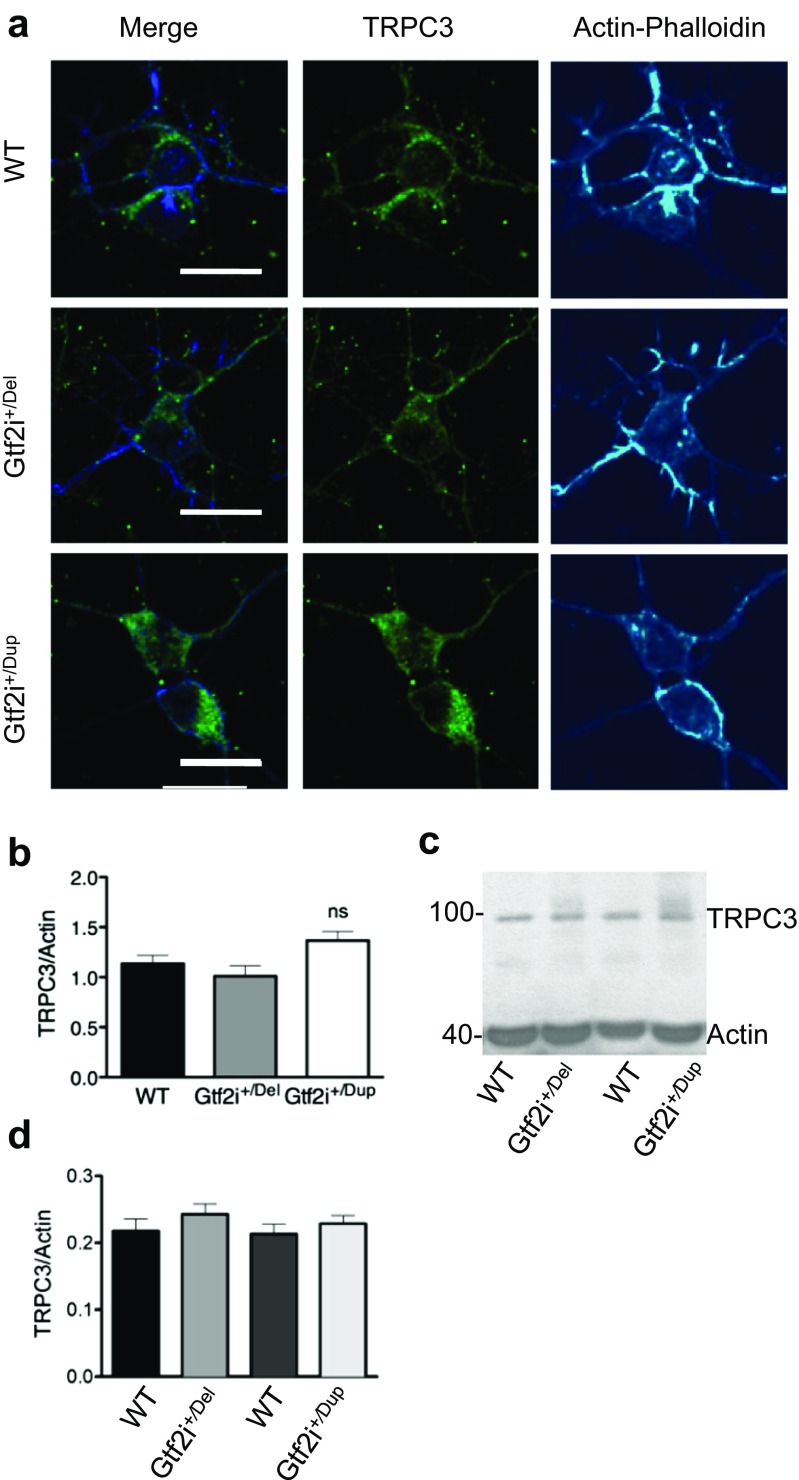
Expression level of TRPC3 in *Gtf2i*^+/Del^ tissue and neurons. **a** Representative confocal images from Div8 neuronal culture of *Gtf2i* mutant mice. TRPC3, green and actin-phalloidin, cyan. Fluorescence intensity was measured using TRPC3/actin ratio and measured in the same area of the cytosol for every cell. **b** Fluorescence intensity summary showed that TRPC3/actin phalliodin intensity did not significantly change between the 3 genotypes. *n* = 40 cells per group from 12 slices from 3 different mice. **c**, **d** Western blot analysis using cortical brain tissue from 7-day-old *Gtf2i* mutant mice; *n* = 6 samples per group.TRPC3 expression level is not significantly different between the WT and samples from their mutant littermates (*P* < 0.005)

**Fig. 4:**
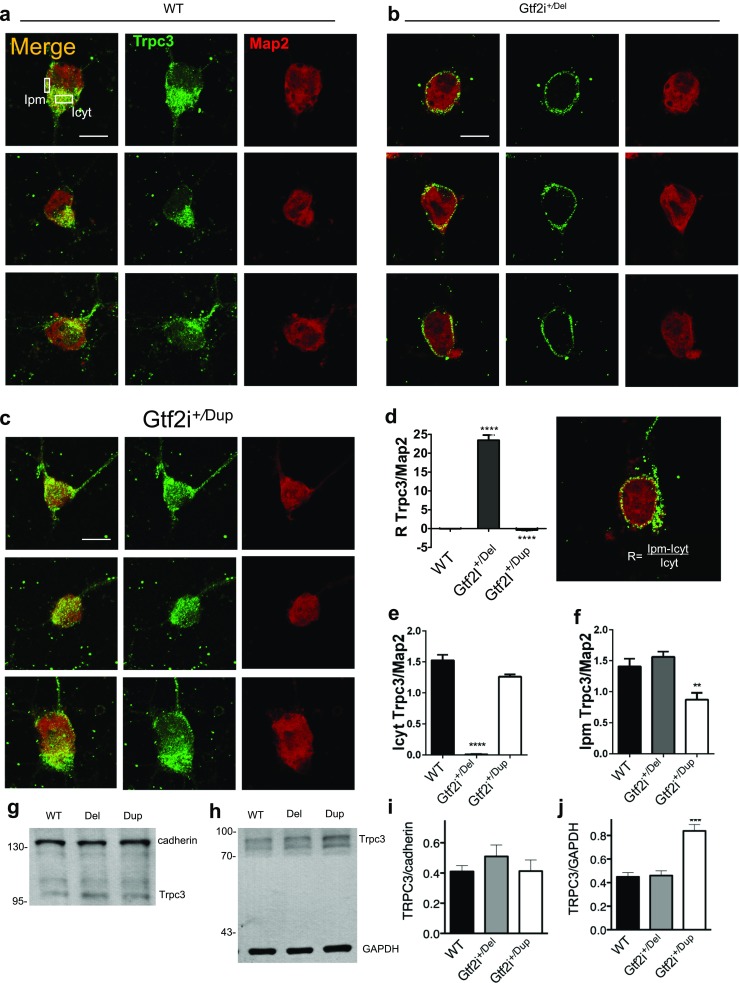
Increased TRPC3 membrane expression in Gtf2i^*+/*Del^ neurons. The cellular localization of TRPC3 in Gtf2i^*+/*Dup^ and Gtf2i^*+/*Del^ cultured cortical neurons was examined using immunofluorescence and confocal imaging. **a**, **b**, **c** Representative confocal images of DIV8 cortical neurons from WT, Gtf2i^*+/*Dup^ and Gtf2i^*+/*Del^. TRPC3, green; MAP2, red. **d**, **e**, **f** Quantitative summary of TRPC3/MAP2 ratio of fluorescence intensity (*n* = 20 for each group). **g** Membrane fraction with cadherin as positive control. **h** Cytosol fraction with GAPDH as control. **i** TRPC3 expression in Gtf2i^*+/*Del^ group is not significantly increased in the membrane fraction comparing with WT and Gtf2i^*+/*Dup^ group, respectively (WT, 0.41 ± 0.038; Gtf2i^*+/*Del^, 0.51 ± 0.076, Gtf2i^*+/*Dup^, 0.41 ± 0.07), *n* = 4. **j** TRPC3 expression in the cytosol fraction is significantly increased in Gtf2i^*+/*Dup^ cortical tissue compared to control (WT, 0.45 ± 0.035; Gtf2i^*+/*Del^, 0.46 ± 0.04, Gtf2i^*+/*Dup^, 0.83 ± 0.054, *P* < 0.0005; *n* = 4). *P < 0.05, **P < 0.005, ***P < 0.0005, indicating the difference to the other groups in the same plot. Scale bar 10 μm

TFII-I has been reported to regulate membrane targeting of TRPC3 channels [12]. We asked whether TRPC3 channels are involved in TFII-I mediated neuronal maturation. We first evaluated the distribution of TRPC3 in DIV8 neuronal cell culture. Figure 3a, b shows confocal immunofluorescence images after immunostaining for TRPC3. TRPC3 fluorescence intensity was not significantly different in either mutant compared to WT. Further, the protein levels of TRPC3 in cortical brain tissues from 7-day-old mice were studied by Western blot analysis. Figure 3c, d shows that the TRPC3 levels were not significantly different between the mutants and their WT littermates. Since altered TFII-I expression can interfere with the correct plasma membrane targeting of TRPC3 in cell lines [12], we anticipated altered membrane expression of TRPC3 channels in *Gtf2i*^*+/*Del^ and *Gtf2i*^*+/*Dup^ cortical neurons as well. Figure 4a–c shows confocal immunofluorescence images of cortical neurons (DIV 8) stained for TRPC3 and MAP2 (neuronal marker). Figure 4d shows the relative translocation ratio (R). Membrane TRPC3 level (over MAP2) in Gtf2i^*+/*Del^ neurons is significantly increased compared to the WT and Gtf2i^*+/*Dup^ (*P* < 0.00005). In contrast, the cytosolic TRPC3 level (Icyt) is significantly decreased in Gtf2i^*+/*Del^ neurons (Fig. 4e) as compared to the WT and Gtf2i^*+/*Del^ neurons (*P* < 0.0005). TRPC3/MAP2 intensity at the plasma membrane (Ipm) (Fig. 4f) is significantly decreased in the WT and Gtf2i^*+/*Dup^ (*P* < 0.005). We further carried out fractionation experiments to investigate the expression of TRPC3 by Western blot in cortical tissue from 7-day-old *Gtf2i* mutant mice (Fig. 4g–j). The expression of TRPC3 in the membrane fraction (Fig. 4g, i) and the cytosol fraction (Fig. 4h, j) was quantified. Cadherin was used as positive control membrane marker and GAPDH was used as cytosol marker. Although expression of TRPC3 in the membrane fraction of Gtf2i^*+/*Del^ tissue was not significantly increased, Gtf2i^*+/*Dup^ samples showed significantly increased expression in the cytosol fractions (*P* < 0.0005, *n* = 4). These results show that the differences in *Gtf2i* copy number and the resulting TFII-I protein levels in Gtf2i^*+/*Dup^ and Gtf2i^*+/*Del^ mice could interfere with correct TRPC3 localization in cultured cortical neurons.

### Altered Ca^2+^ Entry in Gtf2i^*+/*Del^ and Gtf2i^*+/*Dup^ Neurons

As we showed different membrane expressions of TRPC3 in our *Gtf2i* mutant primary neuronal cultures and TFII-I was proposed to function as a negative regulator of intracellular calcium levels by interfering with the transport of TRPC3 to the plasma membrane [12], we studied intracellular calcium levels by stimulating TRPC3 channels. Altered intracellular calcium levels could underlie the decreased neurite outgrowth in *Gtf2i*^+/Dup^ neurons. To stimulate TRPC3 channels, we used carbachol (CCh) which activates the Gq-M1-PLC-DAG-TRPC3 pathway [23]. Calcium levels were measured using ratiometric fura-2 calcium imaging.

**Fig. 5:**
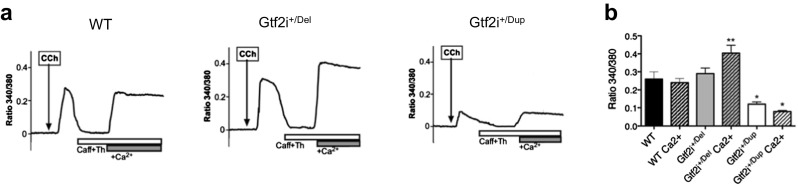
Altered Ca^2+^ entry in Gtf2i^*+/*Del^ and Gtf2i^*+/*Dup^ neurons. **a** Representative fura-2 imaging obtained in the soma of cultured cortical neurons from WT, Gtf2i^*+/*Del^, and Gtf2i^*+/*Dup^ perfused with carbachol (CCh, arrow); additional caffeine and thapsigargin, as indicated by open bar; additional external Ca^2+^ (gray bar, +Ca^2+^). **b** CCh-induced Ca^2+^signals are larger in Gtf2i^*+/*Del^ and smaller in Gtf2i^*+/*Dup^ neurons. *P < 0.05, **P < 0.005, *n* = 25

We first monitored cytosolic fura-2 fluorescence in the absence of extracellular calcium to measure the release from intracellular calcium stores. To fully deplete intracellular stores, we added 10 mM caffeine (an agonist of ryanodine receptors) and 1 μM thapsigargin (to block SERCA pumps; Fig. 5). We then added 2 mM extracellular calcium in the presence of CCh to study calcium entry through plasma membrane channels. As shown in Fig. 5a, b, CCh-induced calcium entry was significantly larger in Gtf2i^*+/*Del^ (*P* < 0.005) and smaller in Gtf2i^*+/*Dup^ (*P* < 0.05) neurons, than in the WT cortical neurons (*n* = 25).

These data collectively provide evidence that TFII-I is a negative regulator of intracellular calcium levels, leading to increased calcium levels in Gtf2i^*+/*Del^ neurons and decreased calcium levels in Gtf2i^*+/*Dup^ neurons. These different intracellular calcium levels could contribute at least in part to altered neurite outgrowth of both *Gtf2i*^+/Del^ and *Gtf2i*^+/Dup^ neurons.

### TFII-I Modulates Ca^2+^ Entry Through TRPC3 Channels

**Fig. 6:**
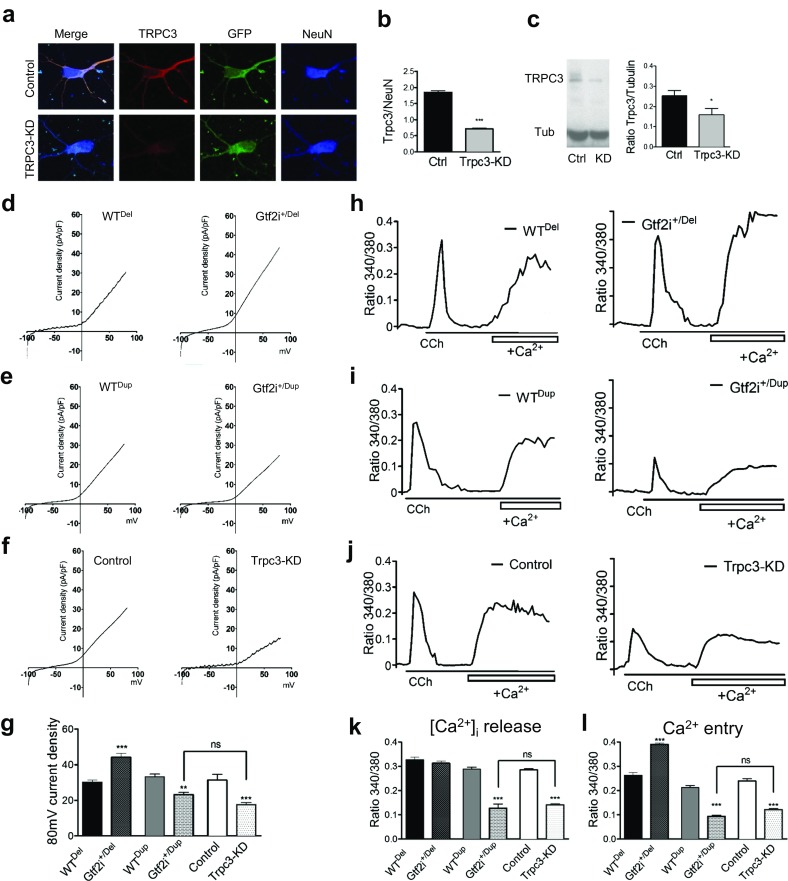
TRPC3 knockdown suppresses Ca^2+^ entry. Cortical neurons were transfected with eGFP-sRNA and *Trpc3*-siRNA-eGFP 4 h after plating. **a** Representatives of confocal images of cortical neurons labeled with TRPC3 in red, GFP in green and NeuN in blue, at DIV4. **b** Quantification of fluorescence intensity of TRPC3 in confocal images shows 40% knockdown of TRPC3 using TRPC3-siRNA in control neurons was confirmed by measuring fluorescence intensity of TRPC3 in confocal images (*P* < 0.0005). **c** Western Blot shows reduction of TRPC3 protein level, confirming TRPC3-knockdown. TRPC3 protein, 97 KDa; β-actin, 42 KDa. (*P < 0.0005). Whole-cell patch-clamp recordings and fura-2 imaging were carried out to study the current density and calcium signals, respectively, in *Gtf2i* mutants and WT neurons with *Trpc3* knockdown. Cortical neurons were transfected with a scrambled-eGFP-siRNA and *Trpc3*-siRNA-eGFP 4 h after plating. Representatives of I-V curves of neurons from *Gtf2i*^+/Del^ mice and their WT littermates designated WT^Del^ (**d**), *Gtf2i*^+/Dup^ and WT^Dup^ littermates (**e**) and WT neurons treated with TRPC3-siRNA-eGFP (Trpc3-KD) or the control vector (**f**). **g** Summary of the whole-cell current density showing a significant increase in *Gtf2i*^+/Del^ neurons (WT^De^ *n* = 10; *Gtf2i*^+/Del^ *n* = 7, *P* < 0.0005), and smaller in *Gtf2i*^+/Dup^ neurons (*n* = 11) compared to their WT littermates (WT^Dup^ *n* = 9, *P* < 0.005). The current density from the neurons treated with TRPC3-siRNA-eGFP was significantly reduced compared to control neurons (eGFP-sRNA *n* = 9; *Trpc3*-siRNA-eGFP *n* = 7, *P* < 0.0005). This decrease in current density of TRPC3-siRNA-eGFP treated cells is comparable and not significantly different to the current density seen in *Gtf2i*^+/Dup^ neurons. **h**, **i**, **j** Ratiometric Fura-2 Ca^2+^ signals measured in the soma. Representative Ca^2+^ traces show carbachol (CCh)-induced Ca^2+^ signal in the absence (showing Ca^2+^ release from internal store) or presence (showing extracellular Ca^2+^ entry) of Ca^2+^ in bath solution. **k** Quantification of [Ca^2+^]_i_ release. WT^Del^ = 86; *Gtf2i*^+/Del^ *n* = 102; WT^Dup^ *n* = 88; *Gtf2i*^+/Dup^ *n* = 88 (*P* < 0.0005); eGFP-sRNA *n* = 80; *Trpc3*-siRNA *n* = 88 (*P* < 0.0005). **l** Quantification of Ca^2+^ entry: WT^Del^ *n* = 86; *Gtf2i*^+/Del^ *n* = 102 (*P* < 0.0005); WT^Dup^ *n* = 88; *Gtf2i*^+/Dup^ *n* = 88 (*P* < 0.005); eGFP-sRNA *n* = 80; *Trpc3*-siRNA *n* = 88 (*P* < 0.0005). *Gtf2i*^+/Dup^, and *Trpc3*-KD are both significantly decreased compared to control. *P < 0.05, **P < 0.005, ***P < 0.0005

To further test if TFII-I modulates changes in Ca^2+^ entry directly through TRPC3 channels, we manipulated TRPC3 levels with specific small interfering RNA (siRNA) and compared them to *Gtf2i*^+/Del^ and *Gtf2i*^+/Dup^ neurons. Validation of TRPC3 knockdown is shown in Fig. 6a–c. We hypothesized that knockdown (KD) of *Trpc3* in cortical mouse neurons would produce an effect resembling the suppressed calcium influx seen in Gtf2i^*+/*Dup^ neurons.

Whole-cell patch-clamp recording was used to test whether current activities of TRPC3-like channels were affected. Representative I–V current curves were showed as Fig. 6d–f. As shown in Fig. 6g, current density in *Gtf2i*^+/Del^ neurons was significantly larger than WT neurons (*P* < 0.0005). In contrast, current density in *Gtf2i*^+/Dup^ neurons was significantly decreased compared to WT and *Gtf2i*^+/Del^ (*P* < 0.005). TRPC3-KD neurons showed a significantly smaller CCh-induced current density at + 80 mV than the eGFP-transfected control neurons (*P* < 0.05). The decrease in TRPC3-KD neurons was comparable and not significantly different than the decrease seen in *Gtf2i*^+/Dup^ neurons.

Next, we carried out Fura-2 calcium imaging to confirm these data (Fig. 6h–j). We tested intracellular Ca^2+^ release, plotted in Fig. 6k, and Ca^2+^ entry plotted in Fig. 6l. CCh-induced Ca^2+^ entry was significantly larger in *Gtf2i*^+/Del^ neurons compared to WT (Fig. 6l, *P* < 0.05). Similar to patch-clamp data, *Gtf2i*^+/Dup^ Ca^2+^ release and entry were significantly smaller than WT (Fig. 6k, l, *P* < 0.0005). Stimulation of TRPC3 channels with CCh in *Trpc3*-targeted siRNA treated neurons resulted in smaller Ca^2+^ signals than that in neurons treated with eGFP-targeted siRNA (*P* < 0.0005) (Fig. 6k, l). These data resemble *Gtf2i*^+/Dup^ properties, supporting our hypothesis that TFII-I modulates Ca^2+^ entry by modulating the activity of TRPC3 channels.

### TRPC3 Knockdown Phenocopies Morphological Phenotype of Gtf2i^+/Dup^ Neurons

**Fig. 7:**
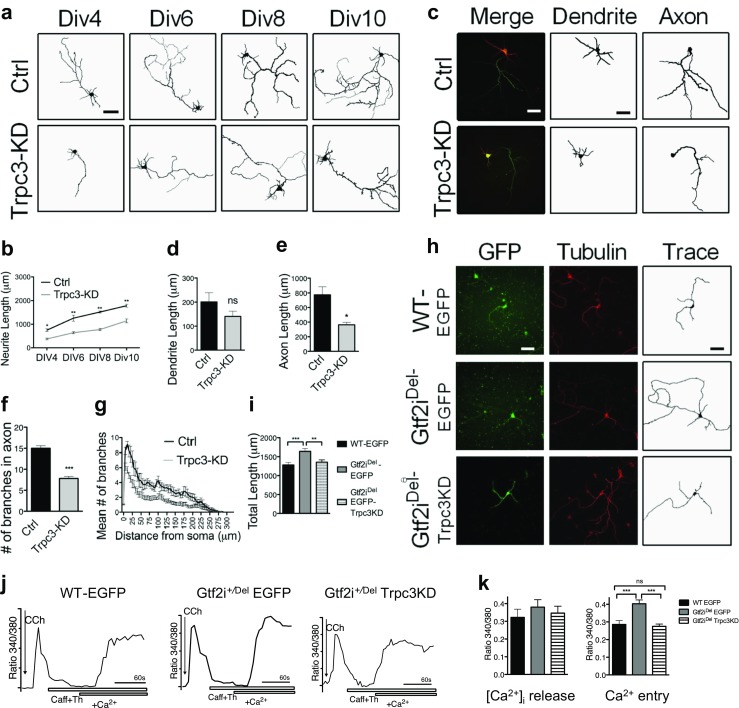
TRPC3 knockdown rescues the phenotype of *Gtf2i*^*+/Del*^ neurons. **a** Representative total neurite traces of neurons at DIV4, 6, 8, and 10. **b** Total neurite length of *Trpc3*-siRNA treated neurons (DIV4 *n* = 125; DIV6 *n* = 95; DIV8 *n* = 80; DIV10 *n* = 86) was significantly shorter than eGFP-sRNA controls (DIV4 *n* = 125; DIV6 *n* = 90; DIV8 *n* = 81; DIV10 *n* = 85, *P* < 0.05). **c** Representative confocal images of neurons immunostained with MAP2 and Tau-1. **d** Quantitative summary of dendritic length in both eGFP-sRNA control group and Trpc3 KD group. eGFP-sRNA *n* = 75; *Trpc3*-targeted siRNA *n* = 70. Unpaired Student’s *t* test. **e** Quantitative summary showed that the axonal length of *Trpc3*-siRNA-treated neurons (*n* = 70) was significantly decreased compared to eGFP-sRNA treated neurons (*n* = 75, *P* < 0.05), unpaired Student’s *t* test. **f** Quantitative summary showed that number of branches in axons was significantly decreased in neurons treated with *Trpc3*-targeted siRNA (*n* = 80) compared to eGFP-sRNA (*n* = 80) treated neurons. (*P* < 0.0005, unpaired Student’s *t* test). **g** Sholl analysis showing Trpc3-KD reduced the number of branches at all the distance measured, as seen in *Gtf2i*^*+/Dup*^. **h** Representative images of WT and Gtf2i^*+/*Del^ neurons immunostained with Tubulin. Total neurite length of GFP positive neurons was traced using the labeling for Tubulin. **i** Summary shows that total neurite length of Gtf2i^*+/*Del^ neurons treated with *Trpc3*-siRNA-eGFP was significantly shorter than control siRNA-eGFP treated Gtf2i^*+/*Del^ neurons (*n* = 40, *P* < 0.005), and similar to control siRNA-eGFP-treated WT neurons. Again, treated with control siRNA-eGFP, the total length of the Gtf2i^*+/*Del^ neurons (*n* = 40) was significantly greater than WT (*n* = 40; *P* < 0.0005). **j** Representative Ca^2+^ traces show carbachol (CCh)-induced Ca^2+^ signal in the absence (showing Ca^2+^ release from internal store) or presence (showing extracellular Ca^2+^ entry) of Ca^2+^ in bath solution. Additional caffeine and thapsigargin, as indicated by open bar. **k** Ca^2+^ release was not significantly different between the 3 groups (*n* = 20). Gtf2i^*+/*Del^ neurons treated with *Trpc3*-siRNA-eGFP calcium entry were rescued to WT levels (*n* = 20; *P* < 0.0005). *P < 0.05, **P < 0.005, ***P < 0.0005

To investigate if the changed Ca^2+^ entry through TRPC3 channels might contribute to the morphological changes seen in Gtf2i^+/Dup^ neurons, we tested if TRPC3 knockdown causes similar morphological changes. To this end, primary cortical neurons were transfected with *Trpc3*-siRNA-*eGFP* or control siRNA-*eGFP*, and the total neurite length, axon length, and axon branching were compared (Fig. 7a–g). *Trpc3*-siRNA-transfected neurons had a significantly decreased total neurite length compared to the controls at all time points (Fig. 7b, *P* < 0.05), similar to that seen in *Gtf2i*^*+/Dup*^ neurons. Dendritic length was similar between *Trpc3*-siRNA-eGFP-transfected neurons and control siRNA-*eGFP*-transfected neurons (Fig. 7c, d), but the axons were significantly shorter in the TRPC3 KD neurons (*P* < 0.0005) (Fig. 7c, e) and less branching was seen (*P* < 0.05) (Fig. 7f). Sholl analysis further indicated that TRPC3 KD diminished the mean number of branches counted at various distances from the soma (Fig. 7g). These results confirmed that the morphological features seen in the TRPC3-KD neurons were similar to those seen in *Gtf2i*^*+/Dup*^ neurons.

### TRPC3 Knockdown Rescues the Phenotype of *Gtf2i*^*+/Del*^ Neurons

To further investigate that the changed Ca^2+^ entry through TRPC3 channels contributes to the morphological changes seen in our mutants, we hypothesized that TRPC3 KD could rescue the phenotype of Gtf2i^*+/*Del^ neurons. Gtf2i^*+/*Del^ primary cortical neurons were transfected with *Trpc3*-siRNA-*eGFP* or control siRNA-*eGFP*, and the total neurite length was quantified (Fig. 7h, i). *Trpc3*-siRNA-transfected Gtf2i^*+/*Del^ neurons had a significantly decreased total neurite length (*P* < 0.005) (Fig. 7h, i), similar to that seen in WT neurons, indicating TRPC3 KD could rescue the morphological Gtf2i^*+/*Del^ phenotype. To assess if the increased Ca^2+^ entry in Gtf2i^*+/*Del^ neurons can be restored to WT levels by TRPC3 KD, we tested intracellular Ca^2+^ release and Ca^2+^ entry in *Trpc3*-siRNA or control siRNA-*eGFP*-transfected Gtf2i^*+/*Del^ neurons (Fig. 7j). While Ca^2+^ release was not significantly different between the groups, CCh-induced Ca^2+^ entry in Gtf2i^*+/*Del^ *Trpc3*-siRNA-transfected neurons had a significantly smaller Ca^2+^ entry compared to control siRNA-*eGFP*-transfected Gtf2i^*+/*Del^ neurons, similar and not significantly different from WT siRNA-*eGFP*-transfected neurons (Fig. 7k).

Taken together, our results demonstrate that knockdown of TRPC3 rescues the morphological phenotype and Ca^2+^ entry levels in Gtf2i^*+/*Del^ neurons, supporting our findings that TFII-I regulates neuronal maturation and intracellular Ca^2+^ levels through the TRPC3 channels.

## Discussion

We have demonstrated that expression levels of TFII-I regulate development of cortical neurons, as well as TRPC3 channel membrane localization. Mice with a single copy of *Gtf2i*, as found in individuals with WS, had increased TRPC3-mediated calcium entry, which coincide with increased axonal length and branching, and increased intracellular calcium levels. In contrast, mice with 3 copies of *Gtf2i*, as found in individuals with Dup7q11.23, had decreased TRPC3 conductance and decreased axon outgrowth and branching. Our findings support the mechanism that the altered copy number of *GTF2I* in WS or Dup7q11.23 results in abnormal axonal outgrowth which may have been caused by a TFII-I mediated regulation of TRPC3 membrane localization.

Our finding that low Ca^2+^ levels suppressed axonal branching in *Gtf2i*^*Dup*^ neurons is consistent with a previous report that a reduced Ca^2+^ influx allows neurite elongation but inhibits axonal branching during development [14]. In addition, we observed that axonal morphology was more affected by Gtf2i levels than the dendrites, when the Ca^2+^ level was altered following manipulating TRPC3 channel. It is well known that the axonal microtubule-binding proteins, Tau, are more sensitive to Ca^2+^-level than the dendritic micotuble-binding protein, MAP2, for microtubule depolymerisation/polymerization dynamics [24]. The differences in axonal and dendritic morphologies in the Gtf2i ^*+/del*^ or in Gtf2i ^*+/dup*^ are therefore likely due to the fine turning of calcium entry via the altered TRPC3.

Activation of muscarinic receptors in neurons promotes axonal outgrowth [15], through PLC-dependent signaling. PLC-γ competes with TFII-I for binding with TRPC3 via its SH3 and PH domains, resulting in membrane translocation of TRPC3 subunits [12], thus increasing the intracellular Ca^2+^ level. In this study, we found that TFII-I did not affect TRPC3 protein expression level, but regulated TRPC3 membrane translocation. Therefore, it is possible that morphological changes, which are due to regulation of TRPC3 translocation by copy number of *GTF2I* in WS and Dup7q11.23, lie downstream of the muscarinic receptor pathway.

The involvement of TRPC3 in neurite outgrowth in cerebellar Purkinje cells had been previously suggested [25]. *Trpc3* null mice lack mGluR-dependent synaptic transmission in Purkinje cells [25], and a gain-of-function *Trpc3* mutation resulted in dramatically impaired growth and differentiation of Purkinje cells [26]. In addition, knockdown of TRPC3 inhibits brain-derived neurotrophic factor (BDNF)-induced turning of growth cones in cultured cerebellar granule cells [27]. BDNF/TrkB signaling increases membrane insertion of TRPC3 and leads to a sustained *I*_BDNF_ current in cultured hippocampal pyramidal cells [28]. The role of TRPC3 in cortical neurons is less well studied. In this study, we show that knockdown of TRPC3 decreased axonal outgrowth in cortical neurons, consistent with the notion that TRPC3 may also be essential for proper neuronal development. The fact that altered TRPC3 expression only regulated axonal development in our study, however, suggests alternative mechanisms of dendritic differentiation in cortical neurons compared to those at play in the cerebellum. Our data support the idea that impaired *Trpc3* conductivity is sufficient for changes in axonal differentiation and morphology to a similar extent as seen in *Gtf2i*^+/Dup^ neurons. This finding adds important new insight beyond the role of TFII-I as a regulator of gene expression [9, 10]; here, we demonstrate that disturbances in key processes of neuronal maturation are partially due to its role in the cytoplasm.

How changes in TFII-I, and subsequently in TRPC3 localization and function, might translate into impaired cognitive performance in adult mice, or humans, remains unknown. Evidence suggests important roles for TRPC3 in early development. TRPC3 presumably plays more important roles in early development than in later stages: in mice, it is homogenously distributed throughout the embryonic cortex [29], whereas in adult animals, it is far more restricted with the most robust expression found in the cerebellum [30]. In humans, TRPC3 protein was found to be expressed over a wide age range in the prefrontal cortex in post mortem tissue, although levels were highest in neonates and infants suggesting a developmental role [31]. We have demonstrated that copy number changes of *Gtf2i* lead to important changes in neuron development and maturation, in particular altered axonal branching which may affect the overall structure or network activity of the mature cortex. Our results support the hypothesis that the changes in TRPC3-mediated calcium influx in the early stages of development may cause a lasting effect in the adult brain.

In conclusion, our study provides evidence that dysregulation of neuronal maturation is induced by differences in TFII-I expression and that this dysregulation is potentially mediated through TRPC3 channel. Our findings provide a starting point to understand the cellular mechanisms that underlie 7q11.23 disorders.

## Electronic Supplementary Material

ESM 1 Supplemental figure S1 Time course showing total neurite length increase in Gtf2i^+/Del^ and decrease in Gtf2i^+/Dup^ neurons compared to WT. Original confocal images were traced and used as representative images at indicated time points showing neurite length in Fig. 1a. Supplemental figure S2 Original confocal images that were traced and used as representative images at indicated time points showing neurite length of *Trpc3-siRNA* treated neurons compared to eGFP-sRNA controls in Fig. 7a. (PPTX 2746 kb)

## References

[1] Mervis CB, Velleman SL (2011). Children with Williams syndrome: language, cognitive, and behavioral characteristics and their implications for intervention. Perspect Lang Learn Educ.

[2] Velleman SL, Mervis CB (2011). Children with 7q11.23 duplication syndrome: speech, language, cognitive, and behavioral characteristics and their implications for intervention. Perspect Lang Learn Educ.

[3] Jackowski AP, Rando K, Maria de AC, Del Cole CG, Silva I, Tavares de Lacerda AL (2009). Brain abnormalities in Williams syndrome: a review of structural and functional magnetic resonance imaging findings. Eur J Paediatr Neurol.

[4] Prontera P, Serino D, Caldini B, Scarponi L, Merla G, Testa G, Muti M, Napolioni V (2014). Brief report: functional MRI of a patient with 7q11.23 duplication syndrome and autism spectrum disorder. J Autism Dev Disord.

[5] Torniero C, Dalla BB, Novara F, Vetro A, Ricca I, Darra F, Pramparo T, Guerrini R (2007). Cortical dysplasia of the left temporal lobe might explain severe expressive-language delay in patients with duplication of the Williams-Beuren locus. Eur J Hum Genet.

[6] Mervis CB, Dida J, Lam E, Crawford-Zelli NA, Young EJ, Henderson DR, Onay T, Morris CA (2012). Duplication of GTF2I results in separation anxiety in mice and humans. Am J Hum Genet.

[7] Sakurai T, Dorr NP, Takahashi N, McInnes LA, Elder GA, Buxbaum JD (2011). Haploinsufficiency of Gtf2i, a gene deleted in Williams syndrome, leads to increases in social interactions. Autism Res.

[8] Fijalkowska I, Sharma D, Bult CJ, Danoff SK (2010). Expression of the transcription factor, TFII-I, during post-implantation mouse embryonic development. BMC Res Notes.

[9] Adamo A, Atashpaz S, Germain PL, Zanella M, D'Agostino G, Albertin V, Chenoweth J, Micale L (2014). 7q11.23 dosage-dependent dysregulation in human pluripotent stem cells affects transcriptional programs in disease-relevant lineages. Nat Genet.

[10] Roy AL (2012). Biochemistry and biology of the inducible multifunctional transcription factor TFII-I: 10 years later. Gene.

[11] Meyer Zu RC, Anastasiadou S, Bachhuber F, Franz-Wachtel M, Macek B, Knoll B (2016). Proteomic analysis of SRF associated transcription complexes identified TFII-I as modulator of SRF function in neurons. Eur J Cell Biol.

[12] Caraveo G, van Rossum DB, Patterson RL, Snyder SH, Desiderio S (2006). Action of TFII-I outside the nucleus as an inhibitor of agonist-induced calcium entry. Science.

[13] Hui K, Fei GH, Saab BJ, Su J, Roder JC, Feng ZP (2007). Neuronal calcium sensor-1 modulation of optimal calcium level for neurite outgrowth. Development.

[14] Gomez TM, Zheng JQ (2006). The molecular basis for calcium-dependent axon pathfinding. Nat Rev Neurosci.

[15] Turlova E, Bae CYJ, Deurloo M, Chen W, Barszczyk A, Horgen FD, Fleig A, Feng ZP (2016). TRPM7 regulates axonal outgrowth and maturation of primary hippocampal neurons. Mol Neurobiol.

[16] Sun HS, Jackson MF, Martin LJ, Jansen K, Teves L, Cui H, Kiyonaka S, Mori Y (2009). Suppression of hippocampal TRPM7 protein prevents delayed neuronal death in brain ischemia. Nat Neurosci.

[17] Nejatbakhsh N, Guo CH, Lu TZ, Pei L, Smit AB, Sun HS, van Kesteren RE, Feng ZP (2011). Caltubin, a novel molluscan tubulin-interacting protein, promotes axonal growth and attenuates axonal degeneration of rodent neurons. J Neurosci.

[18] Barszczyk Andrew, Sun Hong-Shuo, Quan Yi, Zheng Wenhua, Charlton Milton P., Feng Zhong-Ping (2014). Differential Roles of the Mevalonate Pathway in the Development and Survival of Mouse Purkinje Cells in Culture. Molecular Neurobiology.

[19] Schmitz SK, Hjorth JJ, Joemai RM, Wijntjes R, Eijgenraam S, de Brujin B, Georgiou C, de Jong AP (2011). Automated analysis of neuronal morphology, synapse number and synaptic recruitment. J Neurosci Methods.

[20] Meijering E, Jacob M, Sarria JC, Steiner P, Hirling H, Unser M (2004). Design and validation of a tool for neurite tracing and analysis in fluorescence microscopy images. Cytometry A.

[21] Gardzinski P, Lee DW, Fei GH, Hui K, Huang GJ, Sun HS, Feng ZP (2007). The role of synaptotagmin I C2A calcium-binding domain in synaptic vesicle clustering during synapse formation. J Physiol.

[22] Sun HS, Hui K, Lee DW, Feng ZP (2007). Zn2+ sensitivity of high- and low-voltage activated calcium channels. Biophys J.

[23] Massey PV, Bhabra G, Cho K, Brown MW, Bashir ZI (2001). Activation of muscarinic receptors induces protein synthesis-dependent long-lasting depression in the perirhinal cortex. Eur J Neurosci.

[24] Bender PK, Rebhun LI (1986). The calcium sensitivity of MAP-2 and tau microtubules in the presence of calmodulin. Ann N Y Acad Sci.

[25] Hartmann J, Henning HA, Konnerth A (2011). mGluR1/TRPC3-mediated synaptic transmission and calcium signaling in mammalian central neurons. Cold Spring Harb Perspect Biol.

[26] Becker EB, Oliver PL, Glitsch MD, Banks GT, Achilli F, Hardy A, Nolan PM, Fisher EM (2009). A point mutation in TRPC3 causes abnormal Purkinje cell development and cerebellar ataxia in moonwalker mice. Proc Natl Acad Sci U S A.

[27] Li Y, Jia YC, Cui K, Li N, Zheng ZY, Wang YZ, Yuan XB (2005). Essential role of TRPC channels in the guidance of nerve growth cones by brain-derived neurotrophic factor. Nature.

[28] Amaral MD, Pozzo-Miller L (2007). TRPC3 channels are necessary for brain-derived neurotrophic factor to activate a nonselective cationic current and to induce dendritic spine formation. J Neurosci.

[29] Boisseau S, Kunert-Keil C, Lucke S, Bouron A (2009). Heterogeneous distribution of TRPC proteins in the embryonic cortex. Histochem Cell Biol.

[30] Kunert-Keil C, Bisping F, Kruger J, Brinkmeier H (2006). Tissue-specific expression of TRP channel genes in the mouse and its variation in three different mouse strains. BMC Genomics.

[31] Roedding AS, Gao AF, Wu AM, Li PP, Kish SJ, Warsh JJ (2009). TRPC3 protein is expressed across the lifespan in human prefrontal cortex and cerebellum. Brain Res.

