# Supplementary figures and images for: Transcription Factor 2I Regulates Neuronal Development via TRPC3 in 7q11.23 Disorder Models
Source: Mol Neurobiol. 2018 Aug 17;56(5):3313–25. doi: 10.1007/s12035-018-1290-7 (PMC6477017; doi:10.1007/s12035-018-1290-7)

## Slide 1
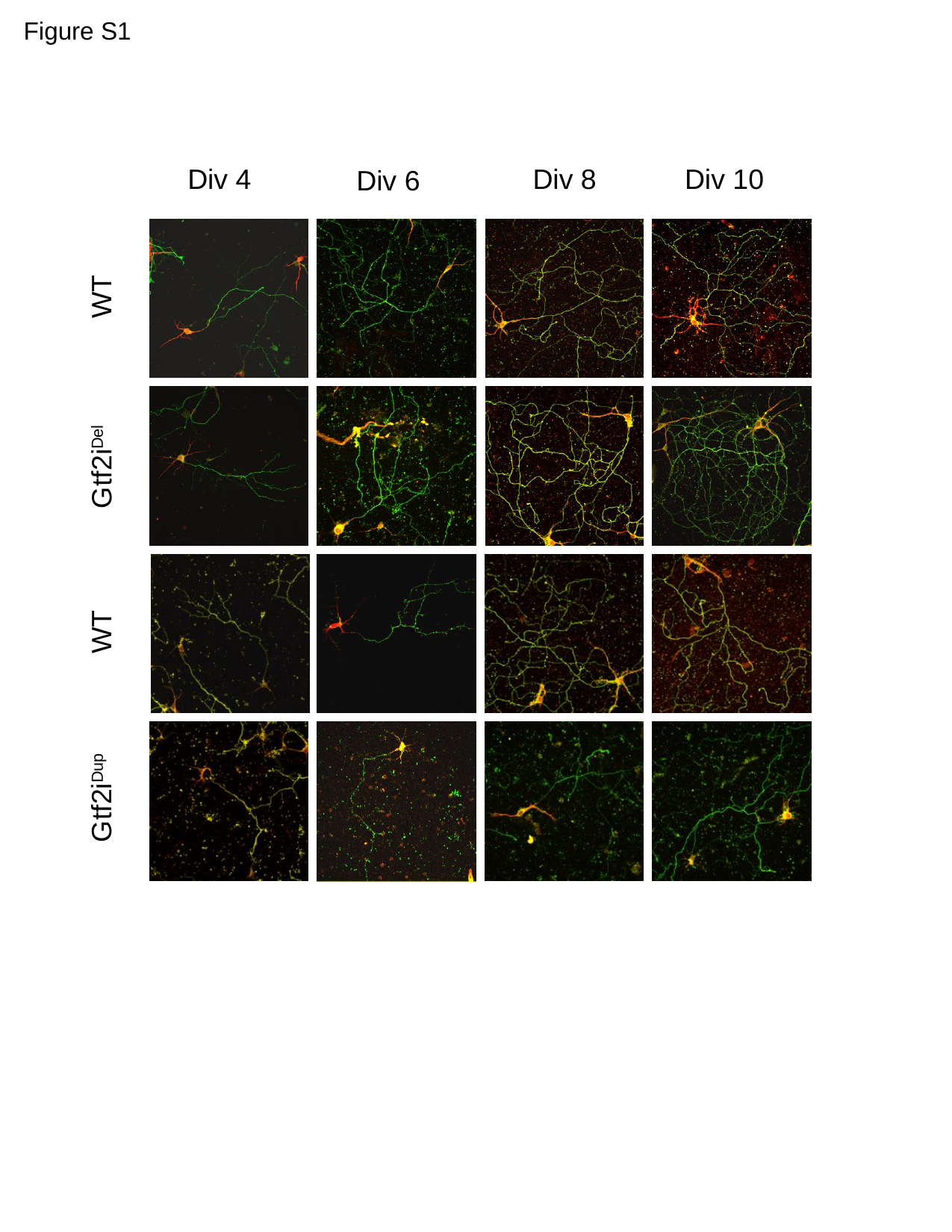

Figure S1
Div 4
Div 8
Div 10
Div 6
WT
Gtf2iDel
WT
Gtf2iDup

## Slide 2
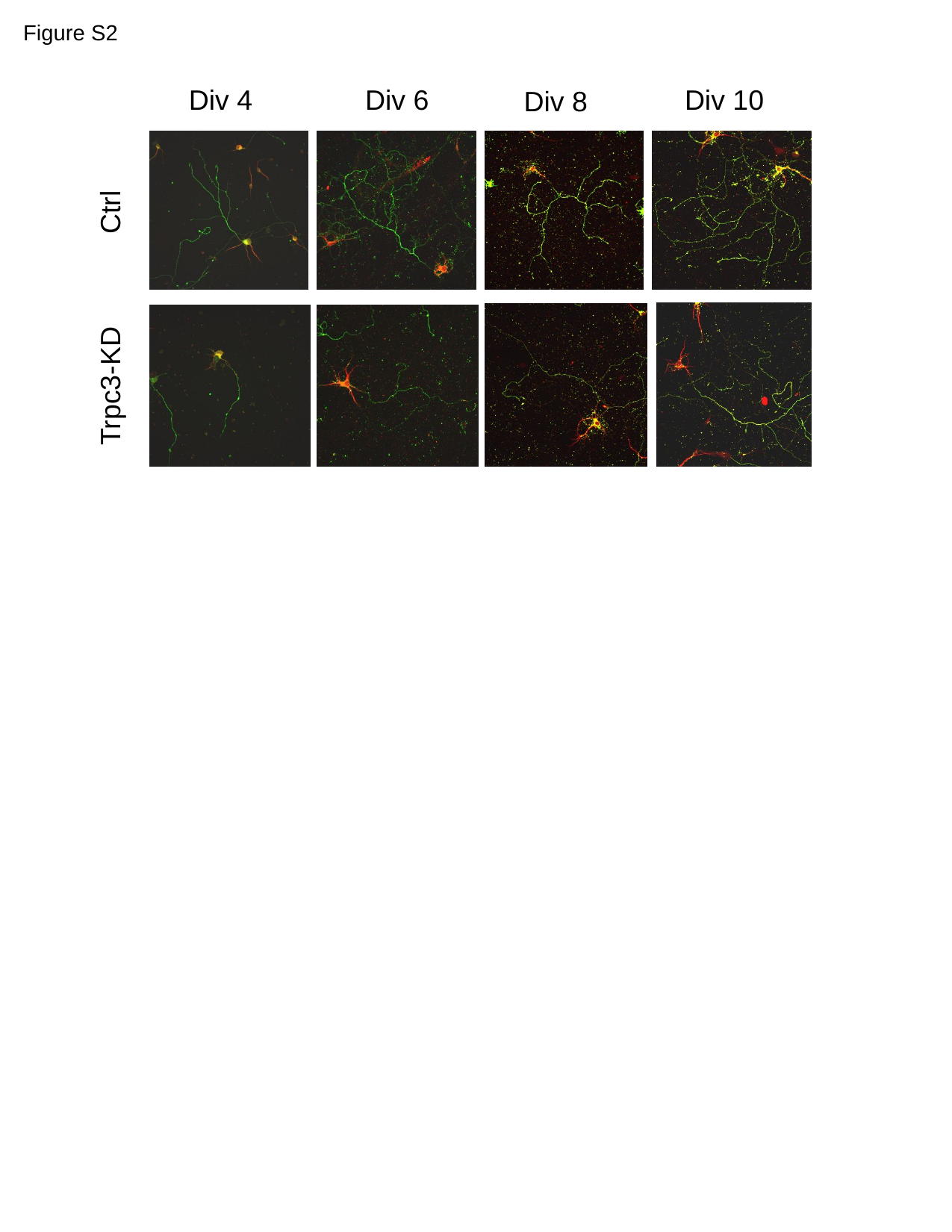

Figure S2
Div 4
Div 6
Div 10
Div 8
Ctrl
Trpc3-KD

Supplement: Supplementary file 1 — Supplemental figure S1 Time course showing total neurite length increase in Gtf2i+/Del and decrease in Gtf2i+/Dup neurons compared to WT. Original confocal images were traced and used as representative images at indicated time points showing neurite length in Fig. 1a. Supplemental figure S2 Original confocal images that were traced and used as representative images at indicated time points showing neurite length of Trpc3-siRNA treated neurons compared to eGFP-sRNA controls in Fig. 7a. (PPTX 2746 kb) [file 12035_2018_1290_MOESM1_ESM.pptx]
